# Hyper-Virulent *Listeria monocytogenes* Strains Associated With Respiratory Infections in Central Italy

**DOI:** 10.3389/fcimb.2021.765540

**Published:** 2021-10-20

**Authors:** Fabrizia Guidi, Alexandra Chiaverini, Antonella Repetto, Cinzia Lorenzetti, Gabriella Centorotola, Viviana Bazzucchi, Barbara Palombo, Antonietta Gattuso, Francesco Pomilio, Giuliana Blasi

**Affiliations:** ^1^ Laboratorio Controllo Alimenti, Istituto Zooprofilattico Sperimentale dell’Umbria e delle Marche “Togo Rosati”, Perugia, Italy; ^2^ Laboratorio Nazionale di Riferimento per Listeria monocytogenes, Istituto Zooprofilattico Sperimentale dell’Abruzzo e del Molise G. Caporale, Teramo, Italy; ^3^ Struttura complessa di Microbiologia, Azienda Ospedaliera di Perugia, Perugia, Italy; ^4^ Dipartimento di Sicurezza Alimentare, Nutrizione e Sanità Pubblica Veterinaria, Istituto Superiore di Sanità, Rome, Italy

**Keywords:** *Listeria monocytogenes*, listeriosis, respiratory infection, whole genome sequencing, LIPI-3, LIPI-4

## Abstract

*Listeria monocytogenes* (*Lm*) is a foodborne pathogen causing listeriosis. Invasive forms of the disease mainly manifest as septicaemia, meningitis and maternal-neonatal infections. *Lm*-associated respiratory infections are very rare and little known. We reported two *Lm* respiratory infection cases occurred in Central Italy during the summer of 2020, in the midst of the SARS-CoV2 pandemic. In addition to collect the epidemiological and clinical characteristics of the patients, we used Whole Genome Sequencing to study the genomes of the *Lm* isolates investigating their virulence and antimicrobial profiles and the presence of genetic mobile elements. Both the strains belonged to hypervirulent MLST clonal complexes (CC). In addition to the Listeria Pathogenicity Island 1 (LIPI-1), the CC1 strain also carried LIPI-3 and the CC4 both LIPI-3 and LIPI-4. Genetic determinants for antimicrobial and disinfectants resistance were found. The CC1 genome presented prophage sequences but they did not interrupt the *comK* gene, involved in the phagosomal escape of *Lm*. None of the strains carried plasmids. *Lm* is an important, although rare, opportunistic pathogen for respiratory tract and lung infections. To avoid dangerous diagnostic delays of these severe clinical forms, it is important to sensitize hospital laboratories to this rare manifestation of listeriosis considering *Lm* in the differential diagnosis of respiratory infections.

## Introduction

*Listeria monocytogenes* (*Lm*) is an important foodborne pathogen causing human listeriosis, a severe disease with high hospitalization and fatality rates when it occurs in invasive form (Camargo et al., 2019). Systemic forms of the disease mainly manifest as septicemia, often complicated by meningoencephalitis in immunocompromised individuals and the elderly; meanwhile, in pregnant women fetal-placental infection can occur, causing abortion and stillbirth (Lecuit, 2020). In addition to these typical presentations, rare forms of localized infections were described and included pleural and lung involvements although very rarely and poorly characterized (Koufakis et al., 2015; Morgand et al., 2018; Lecuit, 2020).

In Central Italy, the national mandatory notification system of invasive listeriosis was integrated with a Laboratory-based Surveillance System, which allows for the collection and typing of the clinical isolates.

Here we present two respiratory tract infection cases associated to *Lm* and occurred in Central Italy during the summer of 2020, in the midst of the SARS-CoV2 pandemic. The aims of this study were to describe the clinical features and the outcome of these rare listeriosis manifestations and to characterize the genomes of the *Lm* isolates using Whole Genome Sequencing (WGS).

## Materials and Methods

### *Lm* Isolation From Body Fluids

The pleural fluid collected from the patient 1 was screened by Gram staining and sub-cultured on different solid media using the BD Kiestra Work Cell Automation automatic system (Becton Dickinson, Sparks, MD, USA) (De Socio et al., 2018). In particular, blood supplemented with colistin and nalidixic acid (CNA), McConkey, mannitol salt, and Sabouraud agar plates were used to detect aerobic bacteria and fungi while Schaedler, and kanamycin-vancomycin (KV) Schaedler agar plates were used for the detection of anaerobic bacteria. Colonies were identified using the MALDI-TOF MS system (bioMérieux, Durham, NC and Bruker Daltonics GmbH, Billerica, MA) (Dingle and Butler-Wu, 2013). The blood culture from patient 2 was performed using the BD BACTEC™ system (Becton Dickinson Diagnostic Instrument Systems, Sparks, Md). Blood samples were collected into two different BACTEC bottles for aerobic and anaerobic culture respectively. Incubation was performed at 34°C-37°C in the Bactec FX instrument for 5 days. Microorganisms’ growth was revealed by the instrument through the CO2 increase detection. All bottles flagged positive were removed immediately from the instrument, and an aliquot was taken for Gram staining and subculture on solid media as described above.

### Antimicrobial Susceptibility Test

Antimicrobial susceptibility test was performed using the Kirby-Bauer disk diffusion method and/or E-test on Muller-Hinton supplemented with horse blood (5%). Results were interpretated according to the European Committee on Antimicrobial Susceptibility Testing (EUCAST) breakpoints (EUCAST v.11.0, 2021).

### *Lm* Strains Collection and Whole Genome Sequencing (WGS) Analysis

Once the diagnosis was confirmed by the hospital laboratory, notification was sent to the Regional Health Authority and then to the Italian Ministry of Health. The *Lm* isolates were sent to the Istituto Zooprofilattico Sperimentale dell’Umbria e delle Marche (IZSUM), as Regional Reference Centre. The strains were typed using WGS in collaboration with the National Reference Laboratory for *Listeria monocytogenes* of Istituto Zooprofilattico Sperimentale dell’Abruzzo e del Molise (IZSAM).

More in detail, DNA extraction was performed using QIAamp DNA Mini Kit (Qiagen Hilden, Germany) according to the manufacturer’s protocol with minor modifications according to Portmann et al. (2018). The purity of the extracts was evaluated by NanoDrop2000 (ThermoFisher Scientific, Wältham, MA). Starting from 1 ng of input DNA, the Nextera XT DNA chemistry (Illumina, San Diego, CA) for library preparation was used according to the manufacturer’s protocols. WGS was performed on the NextSeq 500 platform (Illumina, San Diego, CA) with the NextSeq 500/550 mid output reagent cartridge v2 (300 cycles, standard 150-bp paired-end reads).

For the analysis of WGS data, an in-house pipeline (Cito et al., 2018) was used which included steps for trimming (Trimmomatic v0.36) (Bolger et al., 2014) and quality control check of the reads (FastQC v0.11.5). Genome *de novo* assembly of paired-end reads was performed using SPAdes v3.11.1 (Bankevich et al., 2012) with default parameters for the Illumina platform 2 × 150 chemistry. Then, the genome assembly quality check was performed with QUAST v.4.3 (Gurevich et al., 2013).

The genome assemblies were deposited at DDBJ/ENA/GenBank under the BioProject PRJNA728839 (https://www.ncbi.nlm.nih.gov/bioproject/PRJNA728839), accession numbers JAHAUX000000000 (*Lm*_2510) and JAHAUW000000000 (*Lm*_2541).

Different tools available on the BIGSdb-Lm database (https://bigsdb.pasteur.fr/listeria) were used to deduce *in silico* the seven-gene MLST profile (Salcedo et al., 2003), the Sequence Type (ST) and the Clonal Complex (CC) and to screen the assembled genomes for presence/absence of virulence genes, antimicrobial resistance determinants, Stress Survival Islands (SSI) and disinfectants resistance genes (accessed on 19-03-2021). The presence of Premature Stop Codons (PMSC) in the *inlA* gene was also investigated using the BIGSdb-Lm database (accessed on 19-03-2021). Prophage and plasmids analysis were performed using PHASTER (https://phaster.ca/) (Arndt et al., 2016) and PlasmidFinder 2.0.1 (https://cge.cbs.dtu.dk/services/PlasmidFinder/) (Carattoli et al., 2014) respectively(accessed on 24-03-2021).

## Results

### Case 1

An elderly man presented to the emergency department with exertion dyspnea and chest pain. A nasopharyngeal swab specimen was collected from the patient and tested for the detection of SARS-CoV-2 by RT-PCR, obtaining a negative result. After a chest X-ray, the patient was hospitalized with the diagnosis of pleural empyema. He had a medical history of metastatic right lung adenocarcinoma and he was concomitantly suffering from various pathologies such as: systemic arterial hypertension, benign prostatic hypertrophy, chronic obstructive pulmonary disease, type 2 diabetes mellitus, chronic kidney disease and bilateral hearing loss. He was under pembrolizumab treatment and took a background therapy including insulin lispro, apixaban, ramipril, tamsulosin and pantoprazole. Before the hospitalization the respiratory symptoms were unsuccessfully treated with amoxicillin.

A pleural drainage culture was performed after hospitalization and it returned positive for *Lm;* no other pathogenic organism was isolated from the specimen.

The diagnosis of listeriosis was in accordance with the EU case definition established by the Commission Implementing Decision (EU) 2018/945, meeting both the clinical criterion of localized infection and the laboratory one of *Lm* isolation from a normally sterile site.

The microorganism was tested for antimicrobial susceptibility resulting susceptible to ampicillin, erythromycin, imipenem, meropenem and trimethoprim-sulfamethoxazole according to the breakpoints set by (De Socio et al., 2018) EUCAST. Therefore, during his hospital stay, the patient was treated with the following drugs in addition to the specific background therapy: amoxicillin clavulanate, dexamethasone sodium phosphate, paracetamol, prednisone, trimethoprim-sulfamethoxazole, ampicillin-sulbactam, gentamicin sulfate.

The clinical picture improved and the patient was discharged with the prescription of a therapy based on amoxicillin clavulanate and trimethoprim-sulfamethoxazole.

### Case 2

A female patient presented to the emergency department manifesting fever and dyspnea. She was pregnant in the eighth month of pregnancy, she was no affected by any disease except for mild aortic insufficiency and she did not take any drugs. The SARS-CoV-2 detection RT-PCR test, on the nasopharyngeal swab specimen, resulted negative. The woman was hospitalized with an ultrasound diagnosis of pneumonia. A blood sample was collected and cultured. The culture tested positive for *Lm* with no other pathogenic organism isolated and resulting in a diagnosis of listeriosis (EU 2018/945).

The isolated strain was tested for antimicrobial susceptibility resulting susceptible to ampicillin, meropenem and penicillin G and resistant to erythromycin and trimethoprim-sulfamethoxazole according to the breakpoints set by EUCAST. During hospitalization the patient was treated with clarithromycin, ampicillin-sulbactam, ceftriaxone, metronidazole and paracetamol. Specific iron supplements for pregnancy were also administered.

The women’s clinical condition improved until complete recovery. Labor was induced in the woman at the end of 41th week by oxytocin. The newborn was in good health. He was observed for one week; blood culture and body surface swabs tested negative. The infant was discharged with normal vital signs and increasing weight.

### *Lm* Isolation and Typing

Two *Lm* strains, one for each patient, were isolated and sent to the IZSUM to be typed. In particular, *Lm*_2510 was from the elderly man (Case 1) and *Lm*_2541 was from the pregnant woman (Case 2). The whole genome of both the strains was sequenced obtaining data in agreement with the quality control thresholds recommended (Timme et al., 2020). The MLST analysis showed that *Lm*_2510 belonged to ST1, CC1 (serogroup IVb) while *Lm*_2541 belonged to ST4, CC4 (IVb).

Sixty-four virulence genes were detected in *Lm*_2510 and 70 in *Lm*_2541. The presence/absence of the main virulence genes is reported in **Table 1**.

**Table 1:** Whole Genome Sequencing analysis of the *Lm* isolates: for each strain serogroup, clonal complex, accession number, presence/absence of virulence genes, resistance determinants and intact pro-phage are reported.

		Lm_2510	Lm_2541
Accession number		JAHAUX000000000	JAHAUW000000000
**Typing**	Serogroup	IV b	IV b
	Clonal Complex (CC)	CC1	CC4
**Virulence genes**	LIPI-1	+	+
	LIPI-2	–	–
	LIPI-3	+	+
	LIPI-4	–	+
	Internalin genes	*inlA; inlB; inlC; inlE; inlF; inlH; inlJ; inlK*	*inlA; inlB; inlC; inlE; inlF; inlH; inlJ;*
	*agrA*	+	+
	*agrC*	+	+
	*ami*	–	–
	*aut*	–	–
	*bsh*	+	+
	*cheA*	+	+
	*cheY*	+	+
	*cwhA*	+	+
	*dltA*	+	+
	*fbpA*	+	+
	*fur*	+	+
	*gtcA*	+	+
	*hpt*	+	+
	*lap*	+	+
	*lapB*	+	+
	*lgt*	+	+
	*lisK*	+	+
	*lisR*	+	+
	*lntA*	+	+
	*lpeA*	+	+
	*lplA1*	+	+
	*lspA*	+	+
	*oatA*	+	+
	*oppA*	+	+
	*pdgA*	+	+
	*prsA2*	+	+
	*purQ*	+	+
	*srtA*	+	+
	*srtB*	+	+
	*stp*	+	+
	*svpA*	+	+
	*tagB*	–	–
	*vip*	+	+
	*virR*	+	+
	*virS*	+	+
	*aut_IVb*	+	+
	*comK*	+	+
	*gltA*	+	+
	*gltB*	+	+
	*lmo1280*	+	+
	*lmo2470*	+	+
	*lmo2491*	+	+
	*mdrM*	+	+
**Resistance determinants**	Antimicrobial resistance genes	*fosX; lin; norB; sul; tetA_2; tetA_3; tetC*	*fosX; lin; norB; sul; tetA_2; tetA_3; tetC*
	Multidrug Efflux pumps	*mdrl; lde; SugE*	*mdrl; lde; SugE*
**Intact pro-phage**		PHAGE_Lister_LP_030_3 (NC_024384.1)	–

Both the strains presented a full length *inlA* gene, the Listeria Pathogenicity Island 1 (LIPI-1) including *prfA*, *actA*, *hly*, *mpl*, *plcA*, *plcB*, and *iap* and LIPI-3 (*llsA*, *llsG*, *llsH*, *llsX*, *llsB*, *llsY*, *llsD*, *llsP*). *Lm*_2541 also carried the LIPI-4 consisting of the protein sequences LM9005581_70009 to LM9005581_70014.

Both the strains harbored *fosX* (fosfomycin resistance thiol transferase), *lin* (antibiotic ABC transporter ATP-binding protein), *norB* (multidrug efflux pump) and *sul* (dihydropteroate synthases) for fosfomycin, lincomycin, fluoroquinolones and sulfonamides resistance respectively. The tetracyclin resistance genes *tetA*_2, *tetA*_3 and *tetC* were also detected in their genomes (**Table 1**). Moreover, different multidrug efflux pumps determinants were carried by these strains and in particular, *mdrL* and *lde*, conferring resistance to quinolone and macrolides, and *sugE*, conferring resistance to Quaternary Ammonium Compounds (QAC), aminoglycosides, chloramphenicol, erythromycin and tetracyclines (**Table 1**). Neither *Lm*_2510 nor *Lm*_2541 carried a SSI.

Only in the *Lm*_2510 genome, PHASTER identified 3 prophage regions, of which one intact, one incomplete and one questionable. The intact prophage region (PHAGE_Lister_LP_030_3; GenBank accession number NC_024384.1) presented the highest similarity with the Listeria phage LP-030-3, belonging to the morphospecies 2671 of the Siphoviridae. This prophage region was 42.4kb long and included 67 protein sequences flanked by the recombinant attachment sites attL and attR. This prophage did not interrupt the *comK* gene, found to be complete in *Lm*_2510.The PlasmidFinder web Tool (Accessed on 26-03-2021) did not detect plasmids in either strains.

## Discussion

In this study we reported two *Lm* infections associated with respiratory symptoms occurred in Central Italy in the midst of the SARS-CoV2 pandemic. In the context of an unprecedented health emergency, this rare presentation of human listeriosis caused symptoms that were in part potentially confusing with those of SARS-CoV2 infection, making diagnosis more difficult. Therefore, the priority was to exclude for both patients that they were affected by SARS-CoV2 through the analysis of nasopharyngeal swab and imaging tests.

In the first patient, the X-ray showed the presence of a pleural empyema and the diagnosis of listeriosis was made from pleural drainage culture. The man, in addition to a metastatic lung adenocarcinoma, was affected by several chronic diseases and took many drugs including proton pump inhibitor; the immunocompromised conditions of the patient, together with the reduction of gastric acidity by pantoprazole, certainly made him predisposed towards a *Lm* invasive infection (Kvistholm Jensen et al., 2016; Morgand et al., 2018). Morgand et al. (2018) performed a detailed study on 31 patients with *Lm*-associated respiratory infection, reporting that this presentation of the disease, although rare, occurred in older patients and mostly combined two conditions: immunosuppression, including ongoing neoplasia, and pre-existing lung/pleural disease. The patient of Case 1 had both these conditions, since he was affected by metastatic lung adenocarcinoma.

In the female patient, as she was pregnant, the imaging investigation of respiratory symptoms was performed by ultrasound and it indicated the presence of pneumonia. In this second case, the diagnosis of listeriosis was made from blood culture. Pregnant women represent a population at risk for listeriosis since cell-mediated immunity is moderately depressed during pregnancy (Luca et al., 2015). Although the infection may occur at all stages of pregnancy it is most common in the last trimester as occurred in Case 2. Listeriosis is usually asymptomatic in the mother or causes a mild illness with flulike symptoms such as fever, headache, myalgia and backache, commonly preceded by gastro-intestinal signs (Boucher et al., 1984; Janakiraman, 2008; Luca et al., 2015). However, if the bacteremia causes trans-placental infection, as it often does, spontaneous abortion, fetal death or neonatal infection may occur (Sappenfield et al., 2013; Luca et al., 2015). Listeriosis associated with respiratory symptoms is a rare presentation in a pregnant woman and it was rarely reported (Boucher et al., 1984; Sepúlveda-Bajo et al., 2005). Pneumonia, in particular, is generally considered a rare manifestation of human listeriosis, and it is even more so when it occurs in pregnant ladies. This type of symptomatology is usually reported in affected neonates and not in mothers (De Sá et al., 2004; Chan et al., 2018; Jeffs et al., 2020). However, as such forms of listeriosis occurred in the past, one should always consider *Lm* as a possible etiology for respiratory symptoms complicating pregnancy, itself imposing a stress of the respiratory functions of the mother (Boucher et al., 1984).

In our case, since in addition to the blood culture a sputum culture was not performed, we cannot be absolutely sure that *Lm* was the direct cause of woman’s pneumonia. However, although performing sputum and blood culture is recommended in the diagnosis of severe pneumonia, the clinical utility of the sputum culture has been debated with previous authors reporting its low sensitivity (Ewig et al., 2002; Koufakis et al., 2015). The ultrasound revealed small bilateral subpleural thickenings in the mid-basal region of the lungs. Similar lesions together with the pleural involvement were previously reported in *Lm*-associated respiratory infections (Kim et al., 2012; Morgand et al., 2018).

Certainly, the bacterial nature of the respiratory infection was demonstrated by the successful response to the antimicrobial therapy.

Despite all these findings we could not rule out that *Lm* infection together with pregnancy caused in the patient an immunosuppression state leading to a secondary bacterial pneumonia.

A recent study reported that isolates belonging to the CC1 and CC4 (as *Lm*_2510 and *Lm*_2541) caused respiratory listeriosis (Morgand et al., 2018). These CCs were previously defined hypervirulent since they presented high clinical frequency and harboured particular virulence determinants able to enhance human cells invasion (Maury et al., 2016; Hurley et al., 2019; Maury et al., 2019). One of the major *Lm* virulence factors is the Internalin A (inlA). This protein plays a fundamental role in host cells invasion and in particular, in crossing human intestinal barrier during infection (Franciosa et al., 2009). The presence of PMSC in the *inlA* gene was correlated with the inability of the *Lm* isolates to invade Caco-2 cells and so with a less virulence (Franciosa et al., 2009; Su, 2019). In both the isolates the *inlA* gene presented no PMSC encoding for a full length inlA.

*Lm*_2510 and *Lm*_2541 also carried complete LIPI-1 and LIPI-3. LIPI-1 harbors several important genes, including *prfA, plcA, hly, mpl, actA*, and *plcB*, that participate in host invasion and cellular proliferation and it is widely distributed in *Lm* (Chen et al., 2017). LIPI-3, mainly described in lineage I and previously reported in CC1 and CC4, encodes a biosynthetic cluster involved in the production of Listeriolysin S (LLS), a hemolytic and cytotoxic factor conferring a greater virulence to *Lm* (Maury et al., 2016; Chen et al., 2017; Chen et al., 2019; Tavares et al., 2020). LLS is expressed only under oxidative stress conditions and this confers a better ability in terms of phagosome escape. Pathogenicity studies on murine models demonstrated that LIPI-1 and LIPI-3 were, together, responsible for the increased virulence of some strains (Cotter et al., 2008; Vilchis-Rangel et al., 2019). *Lm*_2541 also carried LIPI-4, a six genes cluster, encoding for a cellobiose-type phosphotransferase system, recently identified (Maury et al., 2016). LIPI-4, highly prevalent in CC4, enhances invasion and in particular, neural and placental tropism of *Lm*, resulting in a strong association of this virulence factor with neural and placental infections (Maury et al., 2016; Chen et al., 2019; Chen et al., 2020). Therefore, infection-associated isolates harboring LIPI-4 are typically considered hypervirulent. *Lm*_2541, despite harboring LIPI-4, caused a rather serious infection in the pregnant woman but did not cross the placental barrier. All these findings made Case 2 even more interesting.

*Lm* is susceptible to most clinically relevant groups of antibiotics active against Gram-positive bacteria, except for intrinsic resistance or reduced susceptibility to fosfomycin, older quinolones, sulfamethoxazole, oxacillin, and expanded-spectrum cephalosporins. The first-line therapy for listeriosis is ampicillin or penicillin G, with or without the addition of gentamicin. For beta-lactam-allergic patients, the therapy of choice is trimethoprim-sulfamethoxazole or vancomycin (Mota et al., 2020). Recently, antibiotic resistance among *Lm* isolated from foods and the environment has increased, particularly for those antibiotics commonly used to treat listeriosis (Olaimat et al., 2018). Therefore, monitoring the *Lm* antibiotic resistance profiles is important. Both the studied strains presented the same antimicrobial-resistance genetic profile harboring specific determinants for fosfomycin, lincomycin, fluoroquinolones, sulfonamides and tetracyclin resistance and non-specific efflux pumps (*mdrl* and *lde*) conferring resistance to quinolone and macrolides (Wilson et al., 2018; Matle et al., 2020). All these genes were chromosomal and not plasmid-borne.

Before administering the therapy to the patients, both in Case 1 and Case 2, antimicrobial susceptibility tests were performed *in vitro* on the relative *Lm* isolates. The tested antimicrobials were erythromycin, penicillin, imipenem, meropenem and trimethoprim-sulfamethoxazole. Only *Lm*_2541 showed resistance to erythromycin and trimethoprim-sulfamethoxazole. This indicated that the mechanism encoded by *sul* was expressed in this strain and not in *Lm*_2510 and that some non-specific system for resistance to erythromycin must exist.

In this regard, the *sugE* gene, encoding for a multidrug efflux pump conferring resistance to aminoglycosides, chloramphenicol, erythromycin and tetracyclines, was also detected in both strains. *sugE*, is also known to confer tolerance to QAC, the most commonly used disinfectants in food industry but also in domestic and hospital environments (Tezel, 2015). The detection of a QAC resistance determinant in these *Lm* clinical isolates suggested they may have survived cleaning and sanitation in some food-processing or food-handling environment and then contaminated products that may have been consumed by the patients, although the source of infection was not identified. In general, the detection of QAC resistance pathways in hypervirulent infection-associated *Lm* strains represents an important public health concern.

None of the strains carried a SSI, for tolerance to environmental stresses, consistently with their belonging to serogroup IVb (Keeney et al., 2018).

As previously reported, the presence of prophages could influence virulence and pathogenicity of *Lm*. In particular, many *Lm* strains carry a prophage within the *comK* gene making it non-functional (Rabinovich et al., 2012; Matle et al., 2020). ComK functions as the master transcription activator of the Competence system (Com), shown to be required for efficient phagosomal escape of *Lm* (Rabinovich et al., 2012). An intact *comK* gene was demonstrated to be required for the expression of the *com* genes during *Lm* intracellular growth (Pasechnek et al., 2020). In the *Lm*_2510 the insertion of the intact and non-intact prophages did not interrupt this transcription regulator since an intact and complete *comK* gene was detected in the isolate. Therefore, the presence of these prophages did not affect the strain’s phagosome escape and its virulence.

The presence of prophages in the *Lm* genomes is of great interest and has been studied by several authors (Chen et al., 2017; Kwon et al., 2020; Matle et al., 2020; Yang et al., 2020). Consistently with our results, a recent CC1-specific study by Moura et al. (2020) reported the detection of intact prophages in *Lm*-CC1 strains.

## Conclusions

*Lm* can be responsible of a wide range of clinical symptoms. *Lm*-associated respiratory infections were previously reported in old patients with immunosuppression and pleural/pulmonary diseases, where they reflect the high host vulnerability. Moreover, although very rarely, adult respiratory distress syndromes associated with maternal listeriosis were also reported (Boucher et al., 1984; Sepúlveda-Bajo et al., 2005).

Confirming the above, in this study, we described two *Lm*-associated respiratory infections occurred in a patient affected by neoplasia and in a pregnant woman not transmitting the pathogen to the fetus.

The differential diagnosis of this rare manifestation of listeriosis was made even more complex by the fact that respiratory symptoms occurred during the SARS-CoV2 pandemic. In medicine, a typical case report, merely describes symptoms, signs, diagnosis, treatment and follow-up of a single or few patients presenting unusual or new manifestations of a disease. The added value of this study is the use of WGS to explore the genome of the *Lm* strains isolated from the patients, characterizing them with high discriminating power.

Both the strains belonged to hypervirulent MLST clones and presented, in addition to the conventional LIPI-1, one or two pathogenicity islands known to confer an increased virulence. These results indicated how the virulence characteristics of a strain play a fundamental role in the major invasive forms of listeriosis. Therefore, the clinical manifestation of human listeriosis proved once again to be the result of the complex interaction between the host’s immune level and the pathogen’s virulence profile. Although specific markers known to be associated with pulmonary tropism have not yet been identified, it would be interesting to perform genome-wide association studies on *Lm* strains isolated from cases of human listeriosis to better understand the genetic basis of many pathogenicity phenotypes, including the respiratory symptoms.

The presence of different genetic determinants conferring antimicrobial and disinfectants resistance was of particular concern both in the treatment of the disease and in the prevention of food contamination by these hypervirulent strains in food processing environments.

Concluding, there are several evidences indicating *Lm* as an important, although rare, opportunistic pathogen for pleural infections and pneumonias compromising the health of immunocompromised individuals and complicating pregnancy.

Therefore, it is important to sensitize hospital laboratories to this rare manifestation of listeriosis considering *Lm* in the differential diagnosis of respiratory infections. This could avoid dangerous diagnostic delays of these severe clinical forms of the disease allowing a prompt intervention with drug treatment. Moreover, in order to make the diagnosis more certain, respiratory samples should be cultured together with blood.

## Data Availability Statement

The datasets presented in this study can be found in online repositories. The name of the repository is https://www.ncbi.nlm.nih.gov/genbank/. The Bioproject, biosample (s) and accession number(s) can be found below: https://www.ncbi.nlm.nih.gov/bioproject/728839, https://www.ncbi.nlm.nih.gov/biosample/19104701, JAHAUX000000000; https://www.ncbi.nlm.nih.gov/bioproject/728839, https://www.ncbi.nlm.nih.gov/biosample/19104702, JAHAUW000000000.

## Ethics Statement

No informed consent was needed since it is a descriptive case series; any sensitive personal data (initials, date of birth, hospitals’ names, etc) or details that would enable any reader (including the individual or anyone else) to identify the person have been omitted.

## Author Contributions

All authors listed have made a substantial, direct, and intellectual contribution to the work, and approved it for publication.

## Conflict of Interest

The authors declare that the research was conducted in the absence of any commercial or financial relationships that could be construed as a potential conflict of interest.

## Publisher’s Note

All claims expressed in this article are solely those of the authors and do not necessarily represent those of their affiliated organizations, or those of the publisher, the editors and the reviewers. Any product that may be evaluated in this article, or claim that may be made by its manufacturer, is not guaranteed or endorsed by the publisher.
